# Adr1 and Cat8 Mediate Coactivator Recruitment and Chromatin Remodeling at Glucose-Regulated Genes

**DOI:** 10.1371/journal.pone.0001436

**Published:** 2008-01-16

**Authors:** Rhiannon K. Biddick, G. Lynn Law, Elton T. Young

**Affiliations:** Department of Biochemistry, University of Washington, Seattle, Washington, United States of America; University College Dublin, Ireland

## Abstract

**Background:**

Adr1 and Cat8 co-regulate numerous glucose-repressed genes in *S. cerevisiae*, presenting a unique opportunity to explore their individual roles in coactivator recruitment, chromatin remodeling, and transcription.

**Methodology/Principal Findings:**

We determined the individual contributions of Cat8 and Adr1 on the expression of a cohort of glucose-repressed genes and found three broad categories: genes that need both activators for full derepression, genes that rely mostly on Cat8 and genes that require only Adr1. Through combined expression and recruitment data, along with analysis of chromatin remodeling at two of these genes, *ADH2* and *FBP1*, we clarified how these activators achieve this wide range of co-regulation. We find that Adr1 and Cat8 are not intrinsically different in their abilities to recruit coactivators but rather, promoter context appears to dictate which activator is responsible for recruitment to specific genes. These promoter-specific contributions are also apparent in the chromatin remodeling that accompanies derepression: *ADH2* requires both Adr1 and Cat8, whereas, at *FBP1*, significant remodeling occurs with Cat8 alone. Although over-expression of Adr1 can compensate for loss of Cat8 at many genes in terms of both activation and chromatin remodeling, this over-expression cannot complement all of the *cat8Δ* phenotypes.

**Conclusions/Significance:**

Thus, at many of the glucose-repressed genes, Cat8 and Adr1 appear to have interchangeable roles and promoter architecture may dictate the roles of these activators.

## Introduction

Alteration in gene expression patterns in response to variations in environmental signals is a tightly regulated process. In the yeast *S. cerevisiae* large changes in transcription accompany the diauxic shift, as organisms switch from using glucose as the main carbon source to non-fermentable carbon sources [1]. Many of the genes that are activated upon glucose exhaustion are under the control of the upstream kinase, Snf1, a homolog of the mammalian AMP-activated kinase [2]. Within the Snf1 network there is a wide range of finer control which includes two gene-specific activators, Adr1 and Cat8, that are necessary for the expression of about two hundred of these genes [3]. Broadly, Adr1 regulates genes involved in peroxisomal proliferation, β-oxidation and utilization of non-fermentable carbon sources (pathways involved in metabolism of ethanol, glycerol and lactate) [3] whereas Cat8 regulates gluconeogenic genes and most of the enzymes in the glyoxylate cycle [4]. However both Adr1 and Cat8 are known to work in tandem to obtain complete derepression of some genes, for example *ADH2* and *ACS1* [5].

This overlapping regulation created by this non-redundant pair of activators is a unique system for controlling gene expression. Other pairs of transcription factors in yeast exist, but they do not mimic the situation observed with Adr1 and Cat8. For example, Ace2 and Swi5 are similar because they co-regulate some genes, while acting independently at others, but Ace2 and Swi5 are highly homologous and bind to the same DNA target sequence, unlike Adr1 and Cat8 [4]. Oaf1 and Pip2 form another set of activators that, although there is some Pip2-independent regulation by Oaf1, are structurally very similar and form a heterodimer at most promoters [6]. Msn2 and Msn4 comprise yet another common pair of activators, but these function almost entirely as a unit, and even their own expression is intimately connected (*MSN4* expression depends on Msn2) [7]. Even Cat8 itself shares its binding site (Carbon Source Response Element or CSRE) with Sip4, another zinc cluster transcription factor, although evidence suggests that Cat8 is more important for regulation of CSRE-containing genes than Sip4 [8]. Unlike the pairs mentioned above, which arose by genome duplication, Adr1 and Cat8 are structurally different, independently expressed transcription factors with distinct binding sites. They often bind to the same promoter under activating conditions, and yet their individual contributions to expression of the gene vary widely.

One mechanism for co-regulation is cooperative binding of transcription factors. This seems to occur at a few promoters regulated by both Adr1 and Cat8. At the *ADH2* promoter, Cat8-binding is enhanced in the presence of Adr1, but the reverse is not true [9]. The same situation is observed at a few other promoters but for the most part, their binding seems to be independent.

Another potential avenue for differential contributions to activation is in a transcription factor's ability to recruit coactivators to the promoter. The paradigm for eukaryotic activator-dependent transcription relies on such recruitment: DNA-binding transcription factors provide specificity by binding preferentially to unique sequences, and then recruit a number of coactivators, such as complexes involved in chromatin remodeling and adaptors between activators and polymerase, as well as the actual transcription machinery itself. A number of these coactivators are non-essential and are only recruited to some promoters [10], a possible explanation for the range of dependence on Adr1 and Cat8. The *HO* promoter in yeast is activated by Swi5 and the heterodimer Swi4/Swi6 (SBF). Swi5 binds first and recruits the coactivator Swi/Snf, followed by Swi4/Swi6 binding [11]. Swi5 also recruits Mediator, a multi-subunit complex thought to act as a bridge between gene-specific activators and the holoenzyme, but does not recruit polymerase itself [12]. A variation on this mechanism is seen with Gcn4, which has two activation domains, each with their own functionality [13]. We asked whether or not Adr1 and Cat8 could also operate in a “division of labor” mechanism to co-regulate many genes, and if so, at the genes dependent only on one activator, if that activator then does the work of two recruiters, or if some coactivators are dispensable.

Starting from previous microarray studies [3], we confirmed the dependencies on Adr1 and Cat8 for derepression of a number of glucose-repressed genes. Based on these results, we further refined the subset of genes at which to study coactivator recruitment (*ADH2, ADY2, FBP1* and *JEN1*). We found that although Adr1 and Cat8 share a common ability to recruit coactivators, this inherent property may be mediated through promoter architecture, which dictates the factor that will be the dominant recruiter. We also looked at chromatin remodeling of two promoters, *ADH2* and *FBP1.* The effects on chromatin remodeling at these promoters upon derepression caused by deletion of Adr1 and/or Cat8 correlated with expression data. When Adr1 was over-expressed however, the role of Cat8 in remodeling was abolished at the *ADH2* promoter, and this again was mirrored in expression levels of this high copy Adr1 *cat8Δ* strain. These data support the conclusion that for coactivator recruitment and chromatin remodeling, Adr1 and Cat8 are not intrinsically unique, and that the observed differences between them may arise from differences in promoter context.

## Results

### A subset of glucose-regulated genes exhibit a range of dependence on the two transcription factors Adr1 and Cat8 for expression

Microarray analysis under glucose-limiting conditions in strains deleted for either Adr1 and Cat8 singly or in combination identified over a hundred Adr1-dependent genes and nearly twice that many Cat8-dependent genes, with 30 genes overlapping these sets [3]. We confirmed the dependence on these transcription factors at a number of genes using real-time quantitative PCR (QPCR) from mRNA isolated from a wild-type strain, a *adr1Δ* strain, a *cat8Δ* strain and a *adr1Δcat8Δ* strain (Table 1). *ADH2*, the canonical Adr1-dependent gene, but which is also known to be both bound and regulated by Cat8 [9], showed a strong dependence on both Adr1 and Cat8 for expression. Some genes showed a strong requirement for just Cat8 (*ICL1, MLS1* and *MDH2*), whereas others (*SPG1, CYB2, CTA1, POT1* and *ALD4*) were only dependent on Adr1. Many genes, however, showed a dependence on both Adr1 and Cat8 that varied between these extremes. We note that at these extremes, however, loss of the unnecessary factor (i.e. loss of Adr1 at *MLS1* or loss of Cat8 at *SPG1*) resulted in significantly *higher* than wild-type levels of expression. This may indicate a metabolic requirement for elevated transcription of some genes to compensate for the loss of other gene products resultant in the absence of activator. Of the strongly Adr1-dependent genes, only *SPG1* contains a consensus CSRE, making it unlikely that Cat8 is acting as a direct repressor. All genes tested, even those that are strongly Cat8-dependent, contain Adr1 binding sites, and furthermore, binding of Adr1 has been observed at a number of these promoters ([9] and references therein).

**Table 1:** Expression in activator mutants^a^

	% WT derepressed (SD)			
Gene b	*adr1Δ*	*cat8Δ*	*adr1Δcat8Δ*	Function
*ICL1*	110 (22)	1.5 (0.2)	1.3 (0.6)	Isocitrate lyase
*ADY2*	14 (11)	2.2 (0.4)	0.6 (0.3)	Acetate transporter
*MLS1*	310 (9.3)	2.1 (0.3)	5.5 (0.5)	Malate synthase
*ADH2*	1.0 (0.1)	4.1 (0.3)	0.1 (0.03)	Alchohol dehydrogenase
*FBP1*	58 (22)	4.2 (0.8)	3.3 (0.8)	Fructose 1,6-bisphosphatase
*MDH2*	154 (8.0)	4.8 (0.6)	8.2 (0.7)	Malate dehydrogenase
*ATO3*	11 (4.2)	6.4 (1.6)	1.2 (0.3)	Ammonia transporter
*ACS1*	34 (15)	16.6 (3.6)	4.2 (0.5)	Acetyl-CoA synthase
*FDH*	6.5 (2.0)	20 (5.2)	11 (8.0)	Formate dehydrogenase
*JEN1*	63 (18)	23 (2.6)	8.4 (1.1)	Lactate transporter
*POX1*	4.0 (1.0)	27 (6.0)	7.1 (1.6)	Fatty-acyl coenzyme A oxidase
*FOX2*	22.2	63.0	14.8	Beta-oxidation enzyme
*SPS19*	18.3	75.0	28.3	2,4 -dienoyl-CoA reductase
*CYB2*	37.0	160	71.8	Lactate dehydrogenase
*ALD4*	12 (2.4)	180 (39)	36 (5.3)	Aldehyde dehydrogenase
*SPG1*	28.0	285	123	Uncharacterized ORF
*CTA1*	58.8	371	171	Catalase A
*POT1*	21 (3.9)	410 (52)	180 (46)	Acetyl-CoA C-acyltransferase

a Values are the average of 3 biological samples, each one quantitated in duplicate and normalized to ACT1 values. The standard deviation is shown in parenthesis, except in cases where the samples were pooled prior to qPCR.

b Genes are arranged in descending order of Cat8-dependence

### Expression data is accurately reflected in the observed occupancy of Pol II and TFIIB

An important function of activators is to recruit and stabilize coactivators and the transcription machinery. Recruitment, therefore, was a candidate for differential roles of Adr1 and Cat8. Using chromatin immunoprecipitation (ChIP) we examined this possibility. We narrowed our recruitment studies to four genes, *ADH2, ADY2, FBP1* and *JEN1*, all of which are dependent on both factors but to differing degrees. In addition, *ADH2, ADY2* and *FBP1* have well-characterized promoters.

**Figure 1:**
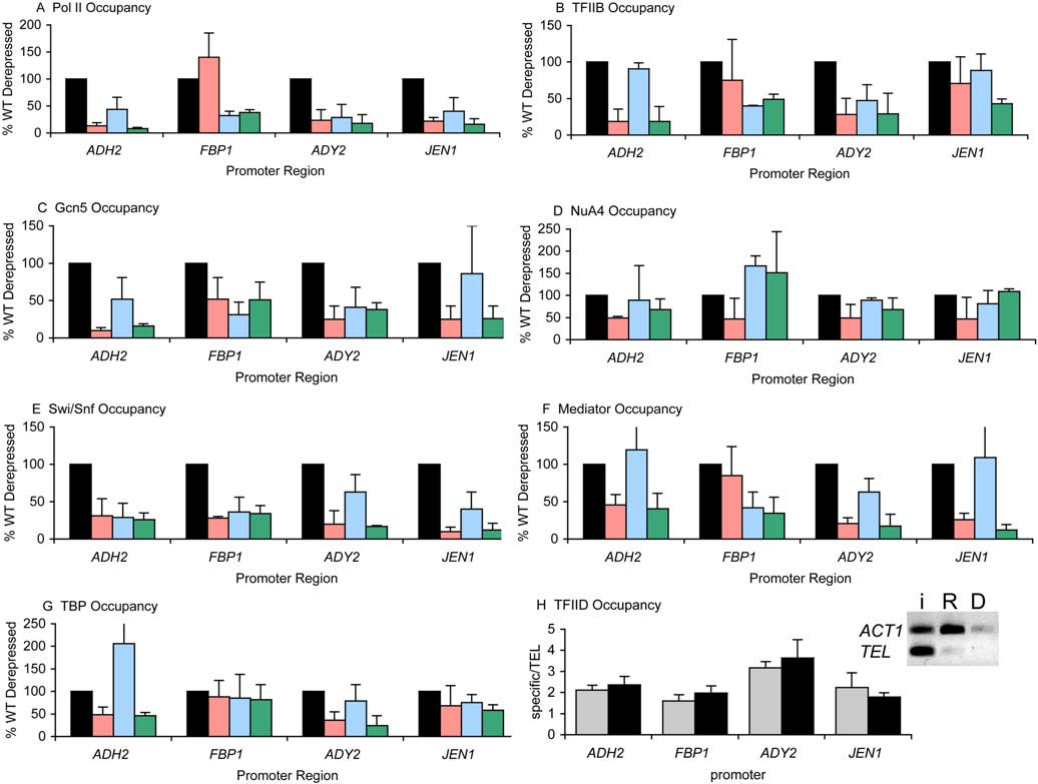
Recruitment profiles under derepressed conditions. A–G: ChIP analysis for coactivators/general transcription machinery at the indicated promoter regions in a wildtype strain (black), *adr1Δ* (pink), *cat8Δ* (blue) and *adr1Δcat8Δ* (green). Binding is expressed as the percent of the wildtype derepressed value (set to 100%) after normalization to the *TEL* negative control locus. Error bars represent the standard deviation of biological replicates (two or more). Data is shown at 4 hours of derepression. H: ChIP analysis for Taf1 in repressing (grey bars) and derepressing (four hours, black bars) conditions. Error bars represent technical replicates. Inset: ChIP analysis by PCR at for Taf1 at *ACT1*. The protein(s) assayed for in each case is listed in Table 2.

We first assayed binding of RNA polymerase II (Pol II). A 5-fold increase on average over the amount of pol II detected at the promoter in repressing conditions was generally observed upon derepression. The amount of pol II at each of the glucose-regulated promoters in repressing conditions was not elevated compared to a region of the telomere not expected to be bound by pol II (additional negative control regions included the coding region of the Pol I structural gene and the *ADH2* ORF yielded the same results). This was true for all of the coactivators we studied (with the exception of TFIID, see below) so data was shown only for derepressed conditions. For mutant strains, the results were expressed as the percent of the factor bound compared to 100% binding in the wild-type strain, both in derepressing conditions. Pol II binding, based on three biological samples, was generally consistent with expression data (compare Fig. 1A and Table 1). The notable exception was the greater than wildtype occupancy at *FBP1* in *adr1Δ* strain. Although Adr1 is not the dominant factor at this gene in terms of expression, it does bind to this promoter [9] and has a minor affect on expression. The increased levels of Pol II in this background may indicate that Adr1 does have a small role and that its deletion requires compensation by increased levels of coactivators.

To further confirm the presence of the Pol II machinery, occupancy at the promoter by TFIIB was determined (Fig. 1B). Again, TFIIB binding is consistent with expression data, although there is some activator-independent binding at *FBP1* and *JEN1*. It is unsurprising that the patterns of Pol II recruitment closely matched those of TFIIB, given their tight association. We did notice, however, that levels of TFIIB occupancy in activator mutants was generally higher than the occupancy of Pol II, which may be an artifact of the ChIP assay (*i.e.* TFIIB cross-links more efficiently), or may represent a more stable association at the promoter, even in the absence of activator. We did not observe either Pol II or TFIIB at any promoter under repressed conditions, as compared to a negative control locus (data not shown).

### Adr1 and Cat8 recruit an array of coactivators upon derepression

We extended our ChIP analysis to study the recruitment of a number of coactivators, in addition to components of the general transcription machinery. To assay occupancy at the promoter, we used epitope tagged representative proteins from each complex (Table 2) to facilitate ChIP analysis in either a wildtype, *adr1Δ, cat8Δ* or *adr1Δcat8Δ* strain. We looked at the recruitment of several chromatin remodeling complexes (SAGA, NuA4 and Swi/Snf), the Mediator complex, and TFIID (Fig 1C–H). In no case did we observe binding at any promoter tested under repressed conditions, and we present here data after four hours of derepression.

**Table 2:** Coactivators used in ChIP Analysis

Protein	Description	Complex
Gcn5^a^	Histone acetyltransferase (HAT)	SAGA, SLIK
Spt8^b^	Involved in histone acetylation	SAGA
Rtg2^b^	Involved in histone acetylation	SLIK
Epl1^b^	Involved in histone acetylation	Nua4
Esa1^a^	Histone acetyltransferase (HAT)	NuA4
Snf2^a^	Catalytic subunit (ATPase-activity)	Swi/Snf
Snf5^b^	Involved in chromatin remodeling	Swi/Snf
Med3^d^	Tail component	Mediator
Med15^c^,^d^	Tail component (Gal11)	Mediator
Med14^c^,^d^	Middle component (Rgr)	Mediator
Med4^d^	Middle component	Mediator
Med18^d^	Head component (Srb5)	Mediator
Med17^c^,^d^	Head component (Srb4)	Mediator
CycC^b^	Cdk/CycC module (Srb11)	Mediator
Taf1^a^	HAT and Ser/Thr kinase activity	TFIID
Taf5^b^	TBP-associated factor	SAGA, TFIID
TBP^a^	TATA-binding protein	TFIID, TFIIB
Sua7^a^	TFIIB	Pol II holoenzyme
Rpb1^a^	Largest subunit of PolII	Pol II holoenzyme

a Results in Figure 1

b Results not shown

c Results are averaged and shown in Figure 1f

d Results shown in Table 3

The requirement for SAGA is not universal at all yeast promoters, as there are reported examples of activated transcription in the absence of both the histone acetyl transferase (HAT) function of SAGA as well as its non-HAT function [14]. Gcn5, the HAT component of SAGA, which is specific for histone H3, was observed at the promoter regions of all four genes tested under derepressing conditions in a wildtype strain. At the *ADY2* and *FBP1* promoters, both Adr1 and Cat8 are required for recruitment, but at *ADH2* and *JEN1*, Adr1 alone sufficed for more than 50% of the recruitment (Fig. 1C). Gcn5 is also a member of the SLIK (SAGA-like) complex [15]. In order to determine whether or not Gcn5 was being recruited as part of SAGA or SLIK, we tagged unique components of both of these complexes and observed the identical pattern of recruitment only with the SAGA-specific protein (Spt8) and not with the SLIK-specific protein (Rtg2) (which was detected at the SLIK-dependent promoter *CIT2*) (data not shown). Esa1, the HAT component of NuA4, which mainly acetylates the H4 tail, bound to promoters upon derepression, however in contrast to SAGA, its recruitment was largely activator independent, suggesting it may play a global, rather than targeted role (Fig. 1D). Similar results were obtained with a second component of NuA4, Epl1 (data not shown).

Regulation of *ADH2* has been reported in the literature to be both dependent on the ATP-dependent remodeling complex Swi/Snf, and independent of it [16], [17]. Our ChIP data for Snf2, the catalytic subunit of Swi/Snf, suggest an activating role for this complex at this subset of genes (Fig. 1E). The recruitment of Swi/Snf was strongly dependent on Adr1 at all promoters tested, and also strongly dependent on Cat8 at *ADH2* and *FBP1*. The contribution to its recruitment by Cat8 is reduced but still observable at *ADY2* and *JEN1*. We also looked at the binding of Snf5 and saw a similar pattern of recruitment (data not shown).

Recent reports on the importance of Mediator reached contradictory conclusions, with some data suggesting Mediator is involved at only a small fraction of yeast genes, and then only when cells are under stress [18], and other data arguing that Mediator is as important as other general transcription factors [19]. It has also been reported that Mediator occupies not only promoter regions, but ORFs as well, and that this occupancy is not necessarily correlated with gene activation [20]. The importance of Mediator under respiratory conditions, however, has never been explored. We first determined the occupancy of Mediator (based on six components, two from each submodule) at both the promoters and ORFs of *ADH2* and *ADH1* (Table 3). *ADH1* is highly expressed under glucose conditions, and down regulated under derepressed conditions. We find that all components of Mediator follow the same pattern of binding, namely, when the gene is active, the complex is found at the promoter. We detected Mediator in the ORF of *ADH1* under repressed (activating) conditions, but at a much lower amount than in the promoter. For the genes we assayed, Mediator occupancy was highest at the promoter under activating conditions.

**Table 3:** ChIP Analysis of Mediator Components^a^

	*ADH1*				*ADH2*			
	Promoter		ORF		Promoter		ORF	
Component	R	D	R	D	R	D	R	D
Med3	6.5	1.5	2.5	0.83	1.6	3.7	0.56	1.1
Med15	7.1	2.1	2.0	0.88	1.3	4.7	0.60	1.5
Med4	9.4	2.4	2.6	1.5	1.1	4.5	0.78	2.3
Med14	11	1.5	2.8	1.1	1.5	3.8	1.0	1.3
Med17	22	3.0	8.6	1.4	0.84	2.1	0.98	0.40
Med18	12	1.5	7.1	2.1	1.6	4.1	1.5	1.9

a Values expressed as the %IP'ed divided by the %IP'ed at the *TEL*

The analysis of Mediator was extended to look at the involvement of activators using three components (Med15 (Gal11), Med14 (Rgr1) and Med17 (Srb4). The patterns of occupancy for these three components were the same and therefore results were averaged together (Fig. 1F). Activator-dependency was expected given the putative role of Mediator as a bridge between transcription factors and general transcription machinery, although at most promoters, one activator by itself was enough to achieve maximal or near-maximal recruitment. Binding was also observed for CycC at *ADH2* under derepressed conditions (data not shown). The presence of CycC/Cdk8, commonly thought of as a repressor [10], along with the rest of Mediator under activating conditions argues against any repressive role of Mediator at these genes.

This subset of genes contains TATA-boxes, so TBP occupancy is expected upon activation. ChIP for TBP reflects this requirement, as a small but significant increase in binding occurs upon derepression. It was difficult to assess the role of activators in this recruitment due to the small range of signal (Fig. 1G). It is clear, however, that Adr1 (and not Cat8) is important for proper recruitment of TBP at *ADH2* and *ADY2*. TBP was expressed from a 2 µ plasmid, and the resulting high levels of TBP may contribute to non-specific binding, accounting for the low specific signals.

ChIP for TFIID, using Taf1 (Taf_II_145) (which was not identified as part of SAGA [21]), suggests that under normal conditions (e.g. in a wildtype strain), these four glucose-repressed genes are TAF-independent, as there is no observable increase in binding upon derepression of a wild-type strain (Fig. 1H). Deletion of activators did not effect the apparent level of Taf1 binding (data not shown). To confirm these results, we looked at the promoter of a gene previously reported to be TAF-dependent, *ACT1* [22], and accordingly saw a large signal under repressed conditions when this gene is more active, and a weaker signal under derepressed conditions when activity decreases (Fig. 1H, inset). Previous reports suggesting that TFIID interacts with Adr1 and is important for *ADH2* expression were based primarily on Taf5 (TAF_II_90) [23], which has since been shown to be a member of both SAGA and TFIID [21], leading to misinterpretation of the importance of TFIID. We detected Taf5 at all four of these promoters, in a similar pattern as Gcn5 (data not shown), further supporting the conclusion that SAGA is required for activation, and TFIID is not.

### Recruitment of these coactivators directly affects expression of this subset of genes

Recruitment of coactivators to a promoter indicates that they play a direct role. However, due to the redundancy of coactivator function, it is important to assess their importance in transcription by gene expression studies. To assess this, we isolated mRNA from strains defective in SAGA, Swi/Snf, or Mediator and looked at expression levels over several hours of derepression, after normalization to *ACT1* (Table 4). The loss of Gcn5 led to reductions in expression of *ADH2* and *FBP1*. Deletion of Ada1, an adaptor protein necessary for the structural integrity of SAGA, led to a more severe diminution of gene expression of *ADH2* and *FBP1*, as well as reductions in expression of *ADY2* and *JEN1* suggesting that SAGA is important for appropriate gene expression both in its capacity as a HAT as well as another, non-HAT function.

**Table 4:** Gene Expression Levels in Coactivator Deletion Strains^a^

	strain				
Gene	*gcn5Δ*	*ada1Δ*	*snf2Δ*	*snf5Δ*	*med17 ts*
*ADH2*	65	3.6	19	1.8	19
*ADY2*	110	10	77	11	5.3
*FBP1*	36	1.7	23	0.27	1.30
*JEN1*	190	47	63	7.0	9.0
*ADR1*	45	61	160	95	110
*CAT8*	65	77	110	88	53

a Values expressed as % wildtype after 4 hours of derepression

Swi/Snf also plays an important role in regulating expression of these genes (Table 4), as shown by the strong reduction in transcription of either a *snf2Δ* or *snf5Δ* strain in the case of *ADH2* and *FBP1*. The effect of the coactivator deletion was less severe at *ADY2* and *JEN1* in the case of *snf2Δ*, but all genes were affected by the loss of Snf5.

Using a temperature sensitive (ts) mutation in Med17, we were able to study the affect of inactivating Mediator. Expression of most of the genes assayed decreased at least 10-fold when Med17 was inactivated (Table 4). *ADH2* was an exception to the near total loss of activity in this strain, with levels still 19% of the wild-type expression. In summary, the expression studies support the conclusions reached from coactivator recruitment. Saga, Swi/Snf1, and Mediator are recruited in a glucose-sensitive manner to the promoters of all four glucose-regulated genes, and all four of them play an important role in transcription.

Because genome-wide studies indicated that the defects caused by loss of these coactivators are widespread [10], we also measured the expression of the activators themselves throughout derepression (Table 4). In large part, transcription levels of Adr1 and Cat8 are unaffected by loss of these coactivators. The slight decreases observed are likely due to a combination of global reduction in transcription and slow growth of these strains, in accordance with the severity of the mutation. Adr1 binding persists at wild-type levels in the *gcn5Δ* and *med17 ts* strains, indicating that although protein levels may be diminished up to 50% compared to a wild-type strain, there is still enough transcription factor to fully occupy the promoter (data not shown).

### Chromatin remodeling at the ADH2 and FBP1 promoters have different requirements for Adr1 and Cat8

**Figure 2:**
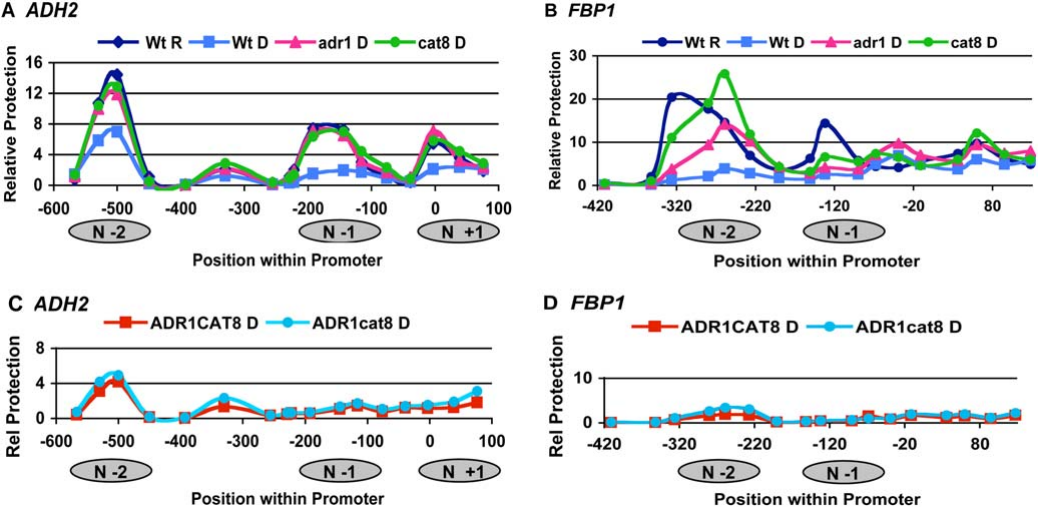
Differential roles for Adr1 and Cat8 in chromatin remodeling at *ADH2* and *FBP1.* NuSA results are displayed as the amount of relative protection, after normalization to the well-positioned nucleosome at *CEN3*. The position of each amplicon (referenced to the middle of each amplicon) within the promoter is shown on the x-axis, with approximate location of nucleosomes shown. (A) and (C) are the results at *ADH2,* (B) and (D) are at *FBP1*. (A) and (B) compare NuSA results of *Δadr1 (*pink) and *Δcat8* (green) in derepressed conditions to a wildtype strain either in repressed (dark blue) or derepressed (light blue) conditions. (C) and (D) compare NuSA results between over-expression of Adr1 with (red) or without Cat8 (blue) in derepressed conditions.

Differential recruitment of known coactivators seems unable to explain the dependence of gene expression on both Adr1 and Cat8. Specific recruitment of unknown coactivators may explain this apparent conundrum. Another possibility is that a step in gene activation requires the simultaneous participation of both Adr1 and Cat8. Previous studies in our lab and others show that specific changes in promoter architecture at both *ADH2* and *FBP1* occur upon derepression and these changes do not occur in a *adr1cat8* double mutant [24], (Infante, unpublished). To determine whether or not the two activators have redundant roles in terms of chromatin structure, we used a nucleosome scanning assay, or NuSA, to measure both nucleosome occupancy and location within the promoter in different yeast strains [25]. Figure 2A shows chromatin remodeling at the *ADH2* promoter in a wild-type strain upon derepression: there is a reduction in protection at all 3 nucleosome positions as predicted [25]. These changes depended on both Adr1 and Cat8, as loss of either one of these activators in derepressing conditions abolished this remodeling. These findings agree with the expression data, in that both activators are required for a substantial increase in expression upon derepression (Table 1). The NuSA at the *FBP1* promoter with a wild-type strain showed changes in chromatin structure upon derepression–a major reduction of protection at N-1 and N-2 and a shift in position of N-2 (Fig. 2B) ([25], Infante unpublished data). However, in this case, differences were seen between the two deletion strains. In the *Δadr1* strain, a significant amount of remodeling occurred. There was a major reduction of occupancy at N-1, occupancy at N-2 was reduced to approximately 50% of repressed levels and the position of N-2 shifted downstream as in wildtype under derepressing conditions. Remodeling in the absence of Cat8, however, was reduced. Some reduction in the N-1 occupancy occurred but less than in the *Δadr1* strain, and the occupancy of N-2 did not change, other than a partial shift in the position. Again the *FBP1* chromatin remodeling agrees with the expression data, in that there is a role for both Adr1 and Cat8, albeit an unequal one.

### Over expression of Adr1 compensates for loss of Cat8 at most genes

The ChIP experiments revealed that both Adr1 and Cat8 are inherently capable of recruiting each coactivator individually (i.e. no coactivator is absolutely dependent on both Adr1 and Cat8 for recruitment) (Fig. 1, and discussion); however, both are necessary for complete chromatin remodeling. This led us to question whether or not they perform unique non-recruitment functions at the promoter, or if there is simply a requirement for a threshold level of activator, achieved at some promoters through a combination of Adr1 and Cat8 and at other promoters largely through only one activator. In order to investigate this possibility, we looked at the levels of activation in strains over-expressing Adr1 in the presence or absence of Cat8. This was done by creating strains chromosomally deleted for either Adr1 or Adr1 and Cat8 together, and then expressing Adr1 from a high-copy plasmid under control of the *ADH1* promoter. At most genes that were dependent on both Adr1 and Cat8, over-expression of Adr1 greatly reduced the requirement for Cat8 (Table 5). Even at the strongly Cat8-dependent genes *ICL1, MLS1* and *MDH2,* there was a reduction in the dependence on Cat8, but not full compensation for its loss. Adr1 does bind to all three of these promoters, suggesting this is a direct effect, despite the fact that normally Adr1 is not required for activation [9] (Table 1). *FBP1* was the sole gene where excess Adr1 cannot alleviate the requirement for Cat8.

**Table 5:** Expression in *cat8Δ* strains with single copy or multi-copy Adr1

	% *CAT8* derepressed (SD)	
Gene	single copy a	multi-copy^b^
*ICL1*	1.5 (0.2)	14 (7.4)
*ADY2*	2.2 (0.4)	75 (47)
*MLS1*	2.1 (0.3)	44 (56)
*ADH2*	4.1 (0.3)	104 (5.3)
*FBP1*	4.2 (0.8)	5.7 (7.2)
*MDH2*	4.8 (0.6)	35 (11)
*ATO3*	6.4 (1.6)	350 (47)
*ACS1*	16.6 (3.6)	160 (48)
*FDH*	20 (5.2)	2900 (670)
*JEN1*	23 (2.6)	1200 (180)
*POX1*	27 (6.0)	830 (180)
*FOX2*	63.0	130 (10)
*SPS19*	75.0	130 (49)
*CYB2*	160	150 (83)
*ALD4*	180 (39)	800 (110)
*SPG1*	285	310 (0)
*CTA1*	371	240 (39)
*POT1*	410 (52)	290 (54)

a Data taken from Table 1 (% WT strain)

b Values are an average of biological duplicates after 4 hours of derepression, normalized to *ACT1* and expressed as the percent of activation in a multi-copy Adr1 *CAT8* strain, with the standard deviation shown in parenthesis.

We used NuSA to ask whether or not the mechanism by which excess Adr1 efficiently activates *ADH2* but not *FBP1* can be explained in terms of chromatin remodeling. Figure 2C shows that the remodeling at the *ADH2* promoter in strains over expressing Adr1 was nearly identical to the remodeling seen in the derepressed wildtype strain, regardless of Cat8, in that protection at N-1 and N-2 was very low compared to protection at *CEN3*. The same was seen at *FBP1* (Fig. 2D), where protection in derepressing conditions was similar to the wild-type levels when Adr1 was over expressed, again regardless of Cat8. The lack of full expression of *FBP1* in the absence of Cat8 cannot be attributed to chromatin remodeling and may be due to missing or reduced levels of coactivators that are normally recruited by this activator.

## Discussion

### A consistent set of coactivators is recruited to and regulates glucose-repressed genes

We have established a set of coactivators that are directly involved in the regulation of glucose-repressed genes based on ChIP and expression studies. In a wild-type strain, recruitment of coactivators did not vary from promoter to promoter. SAGA, Swi/Snf, and Mediator complexes, but not NuA4 or TFIID, are all directly involved in transcription of these genes.

We confirmed the genetic evidence that SAGA plays a role in expression of these genes and extended the analysis to show that it is required in both a HAT and non-HAT capacity. Recruitment of the HAT component Gcn5 (Fig. 1C) demonstrates a direct role for it at the promoter, and the expression data in a *gcn5Δ* strain supports this conclusion (Table 4). The increased severity of the defect in activation seen in the *ada1Δ* strain, however, signifies that SAGA is required beyond just its function as a HAT. Furthermore, the absence of TFIID (Fig. 1H) suggests that the additional role of SAGA is to recruit TBP, a known alternative to its recruitment as part of TFIID [26]. NuA4, the other HAT complex thought to be involved in regulation of these genes, showed weak derepression-dependent binding (∼2 fold increase over background from repressed to derepressed), but it appeared to be largely independent of activators (Fig. 1D). We conclude that it does not have a targeted role. This is supported by other findings of NuA4 as a global regulator of acetylation levels (reviewed in [27]). Esa1, the HAT component, has been previously shown to affect expression of *ADH2* and other glucose-regulated genes [28], in agreement with our ChIP data.

The ATP-dependent chromatin remodeling complex Swi/Snf is part of the set of coactivators required for appropriate expression of glucose-repressed genes. Based on ChIP evidence (Fig. 1E) and the expression data from two separate deletions (*snf2Δ* and *snf5Δ,* Table 2) performed in biological duplicate, we conclude Swi/Snf is involved in direct regulation of *ADH2* and *FBP1*. The variation in expression between *snf2Δ* and *snf5Δ* may reflect a less stringent requirement for this coactivator. Indeed, previous literature gives conflicting results as to the importance of this complex in regulation of *ADH2,* which may have arisen due to differences in derepressing media, time of derepression, and choice of subunit.

In agreement with genome-wide localization studies [10] and recent *in vitro* transcription assays [19], we found an important role for Mediator in activation. Strong binding under derepressed conditions was found for three Mediator components. Our results (Fig. 1F and Table 3) indicate that it is required, as a whole complex (*i.e.* all submodules act in concert) for expression of these genes. Furthermore, we found no evidence of Mediator acting as a repressor.

A recent genome-wide study by Steinmetz et al reported that Pol II occupancy does not strictly correlate with gene activity [29], opening up the question of whether pre-formed preinitiation complexes (PICs) may exist at promoters which are inactive, but poised for fast release from repression, in a similar fashion to what is observed at the heat shock promoters [30]. Despite the relative rapid kinetics of derepression (polymerase can be detected at the promoter as early as ten minutes after glucose depletion (data not shown)), we do not find any evidence for a pre-assembled PIC at these promoters, as neither Pol II nor TFIIB are detectable at promoter regions prior to derepression (Fig. 1A and B).

### Adr1 and Cat8 are not intrinsically unique in their functions

**Figure 3:**
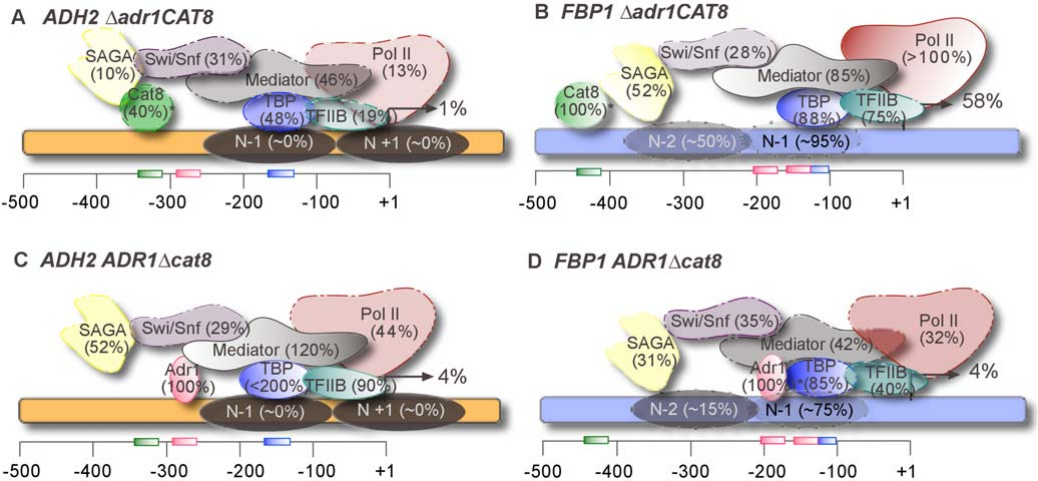
Adr1 and Cat8 play different roles at *ADH2* and *FBP1.* Comparison between the *ADH2* (A and C, orange) and *FBP1* (B and D, blue) promoters in either *adr1*Δ (A and B) or a *cat8*Δ (C and D). Combined expression, recruitment and chromatin remodeling data at 4 hours of derepression are shown. Values in parenthesis indicate the percent binding of the wild-type derepressed value, after normalization (an asterisk represents previously published data [9]). Gene activation is reported as the percent of the wild-type derepressed level. Binding sites are shown at their approximate locations along the promoter (Cat8 in green, Adr1 in pink, and TATA box in blue). Nucleosome positions under derepressed conditions are depicted as shaded ovals on the promoter, and the degree of chromatin remodeling estimated as a percent of the remodeling observed in a wild-type strain. Locations of coactivators are not intended to reflect interactions.

The possibility that these two activators made unique contributions via their ability to recruit different coactivators is ruled out through our extensive ChIP experiments, demonstrating that both Adr1 and Cat8 are capable of recruiting any of the coactivators we studied. Figure 3 summarizes the roles of Adr1 and Cat8 at two contrasting genes, *ADH2* (A and C) and *FBP1* (B and D). The fact that Mediator, for example, is recruited largely by Adr1 at *ADH2*, but by Cat8 at *FBP1* argues that protein-protein interactions are not the governing principle, but rather that it is promoter context that dictates which transcription factor dominates these interactions (see below).

Another possible manner in which they may make distinct contributions to regulation is via chromatin remodeling. This is evidenced by the fact that Adr1 and Cat8 are differentially required for nucleosome remodeling at *ADH2* and *FBP1* (Fig. 3). The non-recruitment function, be it solely through chromatin remodeling or through an unidentified action, may not be very significant, however, in light of our finding that excess Adr1 compensates for the loss of Cat8 in the activation of co-regulated genes (Table 5). At promoters where Cat8 is largely responsible for recruitment (*i.e. FBP1*), excess Adr1 reduces the need for Cat8, but does not abolish it.

Through mass action, high levels of Adr1 may be able to recruit and stably retain more coactivators than it would at wild-type levels. At promoters where Adr1 primarily recruits coactivators (*i.e. ADH2*), Cat8 is dispensable when Adr1 is over expressed, despite what is otherwise a strong requirement for Cat8 in terms of expression. Since the binding sites recognized by Adr1 and Cat8 are very dissimilar, the possibility that stable PIC formation requires the DNA to be bound at two positions or by two separate factors at these promoters is ruled out. Additionally, there is no consistent arrangement of the Adr1 and Cat8 binding sites within promoters of co-regulated genes [9], supporting the conclusion that two separate activator binding events are not necessary for activation of these genes.

Interestingly, we observed that multi-copy Adr1 strains lacking Cat8 fail to grow on glycerol, ethanol, acetate and lactate, all carbon sources whose utilization normally require Cat8 (data not shown) (reviewed in [31] and references therein). These observations suggest that the functional redundancy between these activators appears to be limited to a subset of their target genes, perhaps indicative that they have specialized roles during growth on specific non-fermentable carbon sources. Although we did not perform the converse experiment with over expressed Cat8, we predict that this would yield similar results, with compensation for loss of Adr1 at most genes in this subset, with the exception of those that do not have Cat8 binding sites (*POT1,* for example).

### Promoter architecture may dictate the roles that activators assume at each promoter

In wild-type cells, even at co-regulated genes, subtle differences exist between the roles of Adr1 and Cat8. While they may both share the function of recruiting and stabilizing coactivators, the fact that some promoters display a bias for Adr1 while others depend primarily on Cat8, indicates that under physiological conditions factors exist which dictate which activator dominates regulation (or if both activators will contribute equally). A candidate for such a factor is the promoter architecture, which includes such things as the number and position of nucleosomes, the arrangement of binding sites, and the overall spacing of elements throughout the promoter. For example, it has been demonstrated that the correct helical phasing of Adr1 and Cat8-binding sites is required for wild-type levels of expression of ADH2 [32]. Similar constraints may play a role in the choice of Adr1 versus Cat8 as the more prominent activator at other co-regulated genes.

Nucleosome positioning is another factor that may be important. For example, ADH2 has a repressive chromatin structure under high glucose conditions, with significant remodeling of the nucleosomes upon activation (unpublished data), [28]. The preference for Adr1 at ADH2 may be a result of the fact that the UAS1 is in a nucleosome-free region, whereas at FBP1, the presumed Adr1 binding site is less accessible due to the position of the −1 nucleosome (Fig. 2), and so Cat8, which binds in a nucleosome-free region, becomes the dominant factor.

Based on our studies with these four genes, it appears that despite an overarching shared regulation, the finer workings of the mechanism of activation remain varied. Although there are some commonalities (a consistent set of coactivators), we conclude that the mechanism of activation must be examined on a promoter by promoter basis, even amongst highly related genes. Additionally, the fact that we see no inherent differences in the abilities of Adr1 and Cat8 to recruit coactivators and yet some genes are more heavily dependent on one than the other further supports the conclusion that promoter architecture is equally important for activated transcription.

## Materials and Methods

### Yeast strains and growth of cultures

All strains used in this study were derived from W303. Deletions of activators and epitope tagging of all protein components were introduced according to previously published work [33], [34]. The *med17ts* strain was constructed by first transforming with a *srb4-138*–containing *URA3* plasmid, followed by deletion of chromosomal Med17 according to [33]. TBP was C-terminally 13-Myc tagged and expressed from a 2 µ plasmid. Cultures were grown in YPD (with the exception of strains carrying the TBP plasmid, which were grown in SM lacking leucine for plasmid selection) with either 5% glucose (repressing conditions) or 0.05% glucose (derepressing conditions) at 30°. For the *med17 ts* experiments, cultures were grown overnight at room temperature and then shifted to 37° for 30 minutes before taking the repressed sample; cultures were then spun down in a pre-warmed centrifuge and resuspended in 37° low glucose media and grown for 4 hours. Wild-type strains were grown in the same manner for comparison to eliminate indirect effects of the high temperature. Strains over-expressing Adr1 were created by introducing plasmid YEpNKA1(URA), based on pKD17, which expresses Adr1 from the *ADH1* promoter, with only a minor modification from its original form [35] in a strain chromosomally deleted for Adr1 or both Adr1 and Cat8. For comparison, the single copy Adr1 strain was created in the same strain background using pKD16, a centromeric plasmid carrying a single copy of *ADR1* with its endogenous promoter [35]. These strains were grown in synthetic media lacking uracil or tryptophan, respectively, for plasmid selection with either 5% glucose (repressed) or 0.05% glucose (derepressed).

### ChIP and real-time PCR (QPCR)

ChIP was performed as previously described, using both dimethyladipimate (Pierce chemicals) and formaldehyde as crosslinking agents and using QPCR instead of standard PCR (Tachibana, 2005). We note that the use of an additional cross-linker did make a substantial difference in the observed binding for some proteins. Monoclonal antibodies against c-Myc (9E10)(Santa Cruz Biotechnology sc-40) and HA (F-7)(Santa Cruz Biotechnology sc-7293) epitopes were used for all immunoprecipitations except for Pol II, which was immunoprecipitated using the 8WG16 antibody (Abcam ab817). Primers (IDT) for gene-specific QPCR were designed to cover promoter regions. Sequences are available upon request. QPCR was performed on an MJ Research DNA Engine using Power-SYBR Green (Applied Biosystems). Samples were run in triplicate and relative amounts of DNA were calculated using a standard curve generated from serial dilutions of the input DNA. Standard curves were included for every primer set. Experiments were performed in biological duplicate or triplicate and all results were averaged together. Except where noted, values were calculated as the ratio of the percent immunoprecipitation of ChIP to input at the specific locus to the percent immunoprecipitation at the telomeric region. For repressed samples, this ratio was approximately one. Derepressed values were expressed as the percent of the wild-type value. The associated error results from the standard deviation of the biological replicates. Additional analysis using previously published methods [34], [36] all yielded similar patterns (data not shown).

### mRNA isolation and QPCR

mRNA was isolated from strains grown in either repressing or derepressing media using a hot acidic phenol isolation procedure [37]. The RNA was DNAse I-treated with the Ambion DNAse free Kit and cDNA was made using 1 μg of RNA, oligo dT as primer and SSII-reverse transcriptase (Invitrogen) following the company's procedure. The cDNA solutions were diluted 1∶300 with water before performing QPCR. Gene-specific QPCR was performed in duplicate using primers near the 3′ end of the ORF regions (sequences available upon request). Relative amounts were obtained by comparison to a standard curve and normalized to *ACT1* levels. Samples were prepared from biological triplicates and quantitated in duplicate.

### Nucleosome scanning assay *(NuSA)*

Two hundred mL cell cultures in either repressing or derepressing conditions were processed using the procedures in the yeast culture, micrococcal nuclease digestion, protein degradation and DNA purification steps as outlined in [38]. Specifically, repressed cells were incubated with zymolyase for 15 min. at 30°C while derepressed cells were incubated for 45 min. After the RNAse A digestion, samples were analyzed on 2%-agarose gel to determine the extent of digestions and only samples that were highly enriched in mononucleosomal DNA were gel-extracted using a Qiagen gel extraction kit. DNA samples were diluted 1/300–1/500 and used in QPCR reactions to quantify the presence of a specific amplicon. The protection value set for each amplicon corresponds to the fold-enrichment of that amplicon in the mononucleosomal DNA compared to the undigested-sample and normalized to *CEN3* values. The amplicon used for *CEN3* covered the region shown previously to have a well-positioned nucleosome [39]. QPCR primers were designed to cover the promoter of *ADH2* and *FBP1* with amplicons averaging 100 bp in size (sequences available upon request).
